# Comparison of numerical and verbal rating scales to measure pain exacerbations in patients with chronic cancer pain

**DOI:** 10.1186/1477-7525-8-42

**Published:** 2010-04-22

**Authors:** Cinzia Brunelli, Ernesto Zecca, Cinzia Martini, Tiziana Campa, Elena Fagnoni, Michela Bagnasco, Luigi Lanata, Augusto Caraceni

**Affiliations:** 1 Palliative Care, Pain Therapy, Rehabilitation Unit and 'Virgilio Floriani' Hospice, Fondazione IRCCS, National Cancer Institute of Milan, Milan, Italy; 2 Dompé SpA, Milan, Italy

## Abstract

**Background:**

Numerical rating scales (NRS), and verbal rating scales (VRS) showed to be reliable and valid tools for subjective cancer pain measurement, but no one of them consistently proved to be superior to the other. Aim of the present study is to compare NRS and VRS performance in assessing breakthrough or episodic pain (BP-EP) exacerbations.

**Methods:**

In a cross sectional multicentre study carried out on a sample of 240 advanced cancer patients with pain, background pain and BP-EP intensity in the last 24 hours were measured using both a 6-point VRS and a 0-10 NRS. In order to evaluate the reproducibility of the two scales, a subsample of 60 patients was randomly selected and the questionnaire was administered for a second time three to four hours later. The proportion of "inconsistent" (background pain intensity higher than or equal to peak pain intensity) evaluations was calculated to compare the two scales capability in discriminating between background and peak pain intensity and Cohen's K was calculated to compare their reproducibility.

**Results:**

NRS revealed higher discriminatory capability than VRS in distinguishing between background and peak pain intensity with a lower proportion of patients giving inconsistent evaluations (14% vs. 25%). NRS also showed higher reproducibility when measuring pain exacerbations (Cohen's K of 0.86 for NRS vs. 0.53 for VRS) while the reproducibility of the two scales in evaluating background pain was similar (Cohen's K of 0.80 vs. 0.77).

**Conclusions:**

Our results suggest that, in the measurement of cancer pain exacerbations, patients use NRS more appropriately than VRS and as such NRS should be preferred to VRS in this patient's population.

## Introduction

The importance of pain measurement in routine cancer patient assessment and in research is advocated by experts and scientific associations [1-5], and several efforts are being made to raise consensus on international recommendations in the choice of standardized measurement tools specific for cancer pain evaluation [3,6-8] in both clinical practice and research.

Subjective pain intensity is the most often considered among the dimensions of pain that should be assessed [1], both in the clinic and in clinical trials. Among several subjective methods for pain intensity measurement, visual analogue scales (VAS), numerical rating scales (NRS), and verbal rating scales (VRS) proved to be reliable and valid, but no one of them consistently showed to be superior to the others [9-19]. The three scales are significantly different as to number of response categories, patient and clinician preference, likelihood of missing data and administration requirements [1]. Research consistently shows that the use of VAS in elderly patients is associated with higher failure of completion rates than the use of NRS, and also that the elderly prefer to use NRS in respect to VAS [12,20]. Similar difficulties were observed among patients on high doses of opioids [21]. For these reasons VAS can be considered less suitable for pain evaluation in cancer patients, many of which are old and assume opioids. Yet VAS and NRS have shown a better sensitivity to change with respect to VRS [22] probably due to the usually smaller number of categories in VRS.

For these reasons VAS was not considered in our study, which instead focused on VRS and NRS; both scales are easy to use with most patients and have shown good psychometric properties [22] but no studies have been conducted to compare them for the evaluation of pain exacerbation.

In developing a new questionnaire for breakthrough or intense episodic pain (BP-EP) evaluation, both an 11-point NRS and a 6-level VRS were included in the questionnaire with the aim of comparing their performance in evaluating pain exacerbations in terms of reproducibility and of discriminatory capability to distinguish pain exacerbations over a background of less severe pain.

## Methods

### Patients

This analysis is based on data from 240 patients consecutively enrolled in a cross sectional Italian multicentre study aimed at estimating BP-EP prevalence in a population of advanced cancer patients with pain. The results on prevalence are going to be presented elsewhere. Patients were included if they had a diagnosis of cancer, had cancer-related chronic pain, were at least 18 years of age, and were able to provide written informed consent. Patients were excluded if their pain was exclusively due to a surgical procedure.

### Assessment

The questionnaire for BP-EP evaluation was administered as an interview to the patients by a nurse or a physician; patients were asked to assess their background pain intensity referring to the previous 24 hours and, if they reported to have also episodes of pain exacerbations (both spontaneous or due to volitional or non volitional actions such as movement or cough), they were asked to rate the intensity of their most severe episode during the previous 24. Only for the aims of the present study, the questionnaire for BP-EP evaluation contained a double evaluation both for background pain and for pain intensity exacerbations; one evaluation was performed using a 6-point VRS and patients were asked to rate their pain intensity choosing from the following descriptors: None, Very mild, Mild, Moderate, Severe, Very severe [3]; the second evaluation was performed by an 11 point NRS and patients were asked to rate their pain on a 0 to 10 scale where 0 indicates "No pain" and 10 "The worst possible pain" [1]. This NRS version was chosen from the BPI [23] as the most diffused and validated in Italian language, while the 6-level VRS chosen is a widely used instrument validated across 15 languages [3] which fulfils the requirement of a sufficient number of levels to ensure scale sensitivity [22]. In order to estimate the two scales reproducibility, a randomly selected subsample of 60 patients was administered the questionnaire a second time by a different nurse or physician, three to four hours after the first administration. For the second evaluation the patient was instructed to assess the same 24 hours period already evaluated in the first assessment, excluding the time period between the two administrations.

### Sample size

The sample size of 240 patients was calculated based on the main outcome of the study (prevalence of BP-EP, not reported here). 60 patients were enrolled in the retest phase to ensure a 0.18 precision for the estimates of the reproducibility indexes (where precision indicates the width of the 95% confidence interval). This last calculation was performed in the hypothesis that the reproducibility indexes to be estimated were 0.8 [24].

#### Statistical analysis

The capability of the two scales to discriminate between background pain and pain exacerbations intensities, was measured calculating the proportion of "consistent" and of "inconsistent" evaluations; the evaluation provided by a patient was defined as "consistent" if background pain intensity was lower than peak intensity, otherwise it was defined as "inconsistent" (background pain intensity higher than or equal to peak pain intensity). A higher percentage of inconsistent evaluations on one scale with respect to the other indicates that the former is less adequate for pain exacerbation measurement. The difference between the percentage of inconsistent evaluations obtained through NRS and through VRS, along with its 95% Confidence Interval (95% CI), was estimated to compare the two scales.

Scales reproducibility was evaluated through weighted Kappa (with quadratic weights) and its 95% CI, as a measure of agreement between the first and the second administration of the same scale in the subsample of 60 patients. The strength of the agreement was defined as poor (K < 0.40), moderate (0.41-0.60), substantial (0.61-0.80) and almost perfect (0.81-1.00) [25].

### Ethical approval

The study was approved by the ethics committees of each of the 8 participating centers. It was carried out in accordance with the Declaration of Helsinki, and with Italian laws regarding clinical research. All patients provided written informed consent.

## Results

A study sample of 240 consecutive cancer patients with cancer-related chronic pain (Table 1) was enrolled. About half of them were males, 29% had a cancer of the GI-tract, and 75% had a metastatic disease. Most patients had somatic pain (67%), 40% had neuropathic pain and 158 patients (66%) reported pain exacerbation episodes in the previous 24 hours (Table 2); the most common analgesic medication in the previous 24 hours was a WHO grade III drug (67%). None of the patients screened for eligibility refused to participate in the study and also the compliance to pain evaluations was 100% for both VRS and NRS.

**Table 1:** Clinical characteristics of the study sample (N = 240)

*Characteristic*	*N* *(%)*
Age (years)	
Mean (SD)	61.5 (13.3)
Sex	
Males	126 (52.5)
Females	114 (47.5)
Setting of visit	
Hospital ward	116 (48.2)
Pain therapy outpatients' department	57 (23.8)
Home palliative care	24 (10.0)
Oncology day hospital	24 (10.0)
Hospice	10 (4.0)
Day hospital for pain therapy or palliative care	5 (2.0)
Oncology outpatients' department	4 (2.0)
Primary cancer site or type	
Digestive apparatus^b^	69 (28.8)
Urogenital system^c^	35 (14.7)
Breast	30 (12.5)
Lung	44 (18.3)
Sarcoma	10 (4.0)
Leukemia and lymphoma	5 (2.0)
Head and neck	5 (2.0)
Melanoma	2 (1.0)
Other	40 (16.7)
Extent of disease	
Metastatic	181 (75.5)
Locally advanced	46 (19.2)
Local	5 (2.0)
Unknown	8 (3.3)

^a ^Digestive tract, liver, pancreas

^b ^Ovary, prostate, kidney, uterus, bladder, vulva

^c ^Multiple responses were possible

**Table 2:** Background pain characteristics and analgesic therapy, on the whole sample (n = 240).

*Characteristic*	*N* *(%)*
Pain duration (weeks)	
Mean (SD)	17.7 (21.8)
Type of pain^a^	
Somatic pain	162 (67.5)
Visceral pain	82 (34.2)
Neuropathic pain	97 (40.4)
Pain exacerbations in the previous 24 hours	174 (72.5)
Cause of pain	
The tumor	212 (88.3)
The treatment	6 (2.5)
Other or unknown	22 (9.2)
Anatomical site^a^	
Lower back	60 (25.0)
Abdomen	56 (23.3)
Lower limb	55 (22.9)
Thorax	47 (19.6)
Analgesic medication assumed in the previous 24 h	
None	11 (4.6)
WHO grade 1 (NSAIDs^c^)	14 (5.8)
WHO grade 2	54 (22.5)
WHO grade 3	160 (66.7)
Missing	1 (0.4)

^a ^Multiple responses were possible and only the most common were reported

^c ^NSAIDs: non-steroidal anti-inflammatory drugs

**Figure 1:**
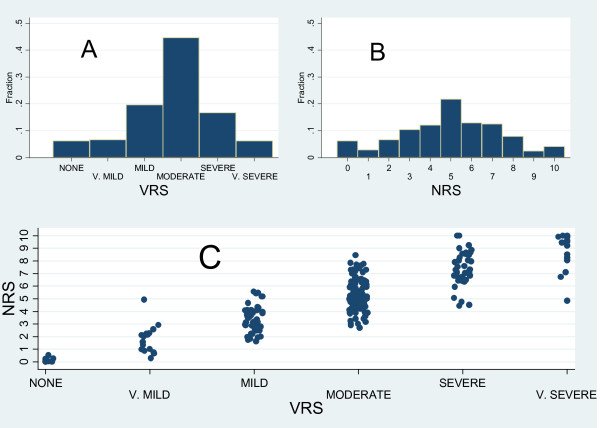
Scores distribution of background pain intensities as measured by VRS (A) and NRS (B) and scatter plot * of the two measures (C). Detailed legend: *In order to avoid an high number of points plotted on top of each other, points have been artificially distributed round their real original position.

Fig. 1A and 1B show the scores distribution of background pain intensities as measured by VRS and NRS respectively, while the same data are shown in a scatter plot (Fig. 1C) which describes the relationship between VRS and NRS scores. The modal value is an intermediate one for both the scales (5 for NRS and "moderate" for VRS) and although a high positive correlation emerged between the two scales (Spearman's rho = 0.86, 95%CI from 0.82 to 0.89), Fig. 1C shows also an high variability in NRS scores at all levels of VRS, especially for "moderate" and "severe" values.

**Figure 2:**
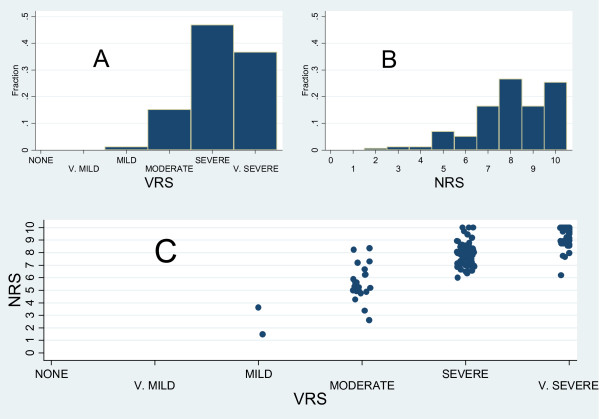
Scores distribution of peak pain intensities as measured by VRS (A) and NRS (B) and scatter plot ^(*) ^of the two measures (C). Detailed legend: ^(*) ^In order to avoid an high number of points plotted on top of each other, points have been artificially distributed round their real original position.

Fig 2A, 2B and 2C illustrate similar data about pain exacerbations evaluation. As expected, the modal values are higher (8 and "Severe" respectively for NRS and VRS) than those for background pain, the correlation between the two scales is positive and high (Spearman's rho = 0.84, 95%CI from 0.79 to 0.88) and the variability of NRS at fixed levels of VRS is reduced.

Table 3 compares the differences between background and peak pain intensities (Δ) for each of the 158 patients who reported to have had pain exacerbations in the previous 24 hours, when using VRS and NRS; when Δ ≤ 0 (i.e. background pain intensity higher or equal to peak pain intensity) the evaluation on that scale is considered inconsistent. Most patients, 116 (73%, 95%CI: 0.66% - 0.80%), gave consistent evaluations on both scales, but a number of them, 42 (27%, 95%CI: 20% - 34%), gave at least 1 inconsistent evaluation. Some patients, 16 (10%, 95%CI: 6% - 16%), gave equal scores (Δ = 0) on both scales: 6 of these patients reported background pain as "very severe" with VRS and as "10" with NRS, being it difficult for them to effectively discriminate between background and peak pain intensities (data not shown in table). 19 patients (12%, 95%CI: 7% - 18%), were not able to discriminate between the intensities of the two types of pain using VRS but managed to do so with NRS, while only 2 (1%, 95%CI: 0% - 4%), patients gave inconsistent NRS and consistent VRS scores. For both scales inconsistency was more likely at higher levels of the baseline pain (data not shown in table). Globally the number of inconsistent evaluations is 23 with NRS (14%, 95%CI: 9% - 21%) versus 40 with VRS (25%, 95%CI: 19% - 33%) with an estimated difference of 11% (95%CI: 5% - 17%) which indicates a significantly higher discriminatory capability of NRS in distinguishing between background and peak pain intensities.

**Table 3:** Comparison of the differences between background and peak pain intensities (Δ) for VRS and NRS on the 158 patients who reported to have had pain flares in the previous 24 hours.

		NRS			
			
		Inconsistent evaluations			
**VRS**		**Δ<0**	**Δ = 0**	**Δ>0**	**Total**

Inconsistent evaluations	Δ<0	2	0	0	2
	Δ = 0	3	16	19	38
	Δ>0	0	2	116	118

	Total	5	18	135	158

Values of Δ less than or equal to 0 on a scale, indicate that background pain intensity was scored higher or equal to peak pain intensity with that scale (inconsistent evaluation).

Table 4 reports the Kappa values for reproducibility evaluation for both scales and for the two different pain intensity measured (background and pain exacerbations) on the 60 patients on which the retest was conducted. The values reported indicate that VRS and NRS have similar reliability when applied to background pain assessment (respectively 0.77 and 0.80) while NRS shows to be more reliable than VRS when measuring pain peaks, with Kappa indexes of 0.86 and 0.53 respectively, which indicate almost perfect versus moderate reproducibility.

**Table 4:** Scales' reproducibility.

SCALE EMPLOYED	TYPE OF PAIN EVALUATED	K	95% CI
VRS	BACKGROUND PAIN	0.77	0.54 - 0.91
	PAIN EXACERBATIONS	0.53	0.20 - 0.77
			
NRS	BACKGROUND PAIN	0.80	0.61 - 0.91
	PAIN EXACERBATIONS	0.86	0.71 - 0.96

## Discussion

This study, comparing NRS and VRS psychometric properties in the assessment of pain exacerbations, reveals a significantly higher discriminatory capability of NRS in distinguishing between background and peak pain intensities referred to the pain experienced in the previous 24 hours; patients gave inconsistent evaluations in 23 cases with NRS (14%) versus 40 cases with VRS (25%). NRS also showed higher reproducibility when measuring pain exacerbations (Cohen's K of 0.86 for NRS vs. 0.53 for VRS) while the reproducibility of the two scales was similar in evaluating background pain (Cohen's K of 0.80 vs. 0.77).

In agreement with previous studies [11,13,26] NRS and VRS showed high positive correlation (Spearman's rho of 0.86 and 0.84 respectively for background and peak pain intensity measurements) although the comparison of the two scales revealed a rather high individual variability mainly for patients scoring "moderate" on the VRS (FIG 1 and FIG 2). This fact suggests that assuming a direct correspondence between VRS and NRS scores (as for example: 0 corresponding to "None", 1-4 to "Mild pain", 5-6 to "Moderate pain", and 7-10 to severe pain1 [14,27-29]), should be interpreted cautiously in clinical practice due to relevant individual discrepancies.

Moreover the wider range of NRS scores at any value of VRS suggests that patients benefit from the greater sensitivity offered by the higher number of response levels possible with NRS. The possibility to increase the number of verbal descriptors in VRS scales has also limitations. A study by Rosier et al. [30] showed that among 15 adjectives offered to describe their pain, on average patients used only 6 of them, perhaps also because of a difficulty in distinguishing and ordering such high a number of verbal descriptors.

In this experience no data were missing for both scales. This is probably due to the fact that the pain evaluations were not self-completed by patients but administered by a trained nurse or physician, who could properly help patients in understanding questions. Some patients who did not give consent may have had physical or cognitive impairment and this could have contributed to increase the compliance with pain assessment. Although good compliance with the use of NRS is confirmed also in the clinical use, the two scales applicability should be verified in different conditions such as self-administration and repeated use in time.

One limit of the study could be that the two scales have different upper anchor descriptors: "The worst possible pain" for the NRS and "Very severe" for the VRS. The two scales formats have been chosen because they both have undergone specific validation studies in Italian and other languages [23,31,32] and fulfill the requirement of a sufficient number of levels to ensure scale sensitivity [22].

The ability of the patient to report his/her pain assessment over the same 24 hours period 3 to 4 hours after the first administration, could be questionable. This choice is aimed to avoid reproducibility overestimation due to memory effect of the first assessment. Furthermore the potential bias introduced by a 3 to 4 hours interval, should have resulted in an underestimation of reproducibility while the indexes obtained (Cohen's K of 0.80 and 0.77 respectively for baseline NRS and VRS) indicate substantial agreement.

In addition, these results should be considered within the limits of the study methods which required the assessment of previous 24 hours pain in a population of advanced cancer patients with no clinically evident cognitive impairment and in relatively good general conditions (38% of patients were out patients and only 14% were admitted to hospice or home care programs).

Previous studies have already compared various scales for pain measurement and gave different results [13-15,18,19,22,26,33-35]. Various factors may have influenced the differences in the results of these studies such as patient's populations (chronic or acute pain, different ages, and different levels of cognitive impairment), types of pain (usual background pain, breakthrough pain), different settings of care (clinical or experimental) and administration methods (self-administration or interview). It s also possible that the lack of agreement on the core properties of the measurement scales and on the analysis methods used to evaluate them, lead to apparently different conclusions depending on the different priority given to various scales properties such as easiness of compilation, validity, sensitivity to change and reliability [11,15,26], appropriateness of linearity assumption [18] or stability of intra-individual assessment [19].

The data from the literature favoring the use of NRS for pain measurement are based on its intrinsic measurement properties [36], its cross-cultural validity [29,37], and its good responsivity properties [38]. Moreover, the high variability of VRS formulations both in the number of response categories and in the labels attached to these categories, support the use of NRS which is applied with more standardized formats (usually 11 levels from 0 to 10) across cultures and languages [3,30,39]. The 0-10 NRS has greater sensitivity than the VRS and achieves an adequate level of discrimination [22]. The use of VRS is usually supported by its easy of administration, mainly in some patient's populations [1,16].

## Conclusion

Our results suggest that in the measurement of cancer pain exacerbations, patients use NRS more appropriately than VRS and as such NRS should be preferred to VRS in this patient's population.

## Abbreviations

(VAS): Visual Analogue Scale; (NRS): Numerical Rating Scale; (VRS): Verbal Rating Scales; (BP-EP): Breakthrough or intense Episodic Pain; (CI): Confidence Interval.

## Competing interest

CB, EZ, CM, and AC have undertaken consultancy work for Dompé SpA. LL and MB are employees of Dompé SpA, Milan, Italy.

## Authors' contributions

CB participated in the design of the study, performed the statistical analysis and drafted the manuscript. EZ, CM, TC, EF, participated in the design of the study, collected data and revised the drafted manuscript. MB, LL participated in the design of the study and revised the drafted the manuscript. AC conceived and coordinated the study, participated in its design and drafted the manuscript. All authors read and approved the final manuscript.
